# Night Work and Social Jet Lag: Pathways to Arterial Stiffness?

**DOI:** 10.3390/clockssleep7010010

**Published:** 2025-03-03

**Authors:** Waléria D. P. Gusmão, Aline Silva-Costa, Victor M. Silva, Claudia R. C. Moreno

**Affiliations:** 1 Center for Integrative Sciences, Alagoas State University of Health Sciences (UNCISAL), Maceio 57010-382, Brazil; waleria.dantas@uncisal.edu.br; 2 Department of Collective Health, Federal University of Triangulo, Mineiro, Uberaba 38025-350, Brazil; costaaline@id.uff.br; 3 Department of Health and Society, School of Public Health, University of Sao Paulo, Sao Paulo 01246-904, Brazil; victor.ms@usp.br

**Keywords:** night work, social jet lag, arterial stiffness

## Abstract

Cardiovascular diseases are the leading cause of morbidity and mortality worldwide. These conditions, characterized by multifactorial etiology, are associated with arterial stiffness, and adequate sleep serves as a preventive factor. Professionals engaged in night work are at an increased risk of premature vascular aging due to potential disruption of the sleep–wake cycle and sleep restriction. The aim of this study was to assess the relationship between duration of exposure to night work and arterial stiffness in nursing professionals. A total of 63 nursing professionals working rotating shifts participated in the study. Arterial stiffness was measured using oscillometric pulse wave velocity, and sleep–wake patterns were monitored using actigraphy. Path analysis revealed no direct association between duration of night work exposure and arterial stiffness in the professionals studied. However, an increase of 1 standard deviation (SD) in social jet lag duration was significantly associated with a 0.212 SD increase in perceived stress (*p* = 0.047). Furthermore, an increase of 1 SD in social jet lag duration was significantly associated with a 0.093 SD increase in the highest pulse wave velocity (*p* = 0.034). Thus, an association was found between increased social jet lag and elevated pulse wave velocity, an independent predictor of higher cardiovascular risk.

## 1. Introduction

The etiology of cardiovascular disease (CVD) is multifactorial, with a number of factors contributing to increased cardiovascular risk, as follows: elevated systolic blood pressure, inadequate dietary habits, a high body mass index, elevated levels of total cholesterol and/or fasting plasma glucose, smoking, and a low level of physical activity [1]. These factors have also been directly linked to early vascular aging, which can be identified by increased arterial stiffness, i.e., high pulse wave velocity, and poor arterial functioning, as determined using elevated augmentation index [2].

In response to the negative impact of CVD on the global population, the American Heart Association (AHA) has identified the following eight key priorities for cardiovascular outcome prevention: diet, physical activity, nicotine exposure, body mass index, serum lipids, blood glucose, blood pressure, and healthy sleep. Of these parameters, sleep is the most recent addition [3], as insufficient or poor-quality sleep is considered a risk factor for all-cause mortality [4] and cardiovascular outcomes [4,5].

Many workers accumulate sleep debt due to chronic sleep restriction imposed by work schedules and social commitments [6]. Observing the misalignment in sleep–wake times between workdays and days off, Roenneberg’s group [7] introduced the concept of social jet lag (SJL) to define misalignment between social time and individual biological temporal markers. SJL is highly prevalent, affecting more than half of the general population, suggesting an even greater prevalence among shift workers [8]. The phenomenon has been associated with numerous negative health consequences, including an increased risk of type 2 diabetes [9], obesity [9], and CVD [10].

The fast-paced society of the 21st century demands continuous work operations 24 h a day, seven days a week, leading to the widespread adoption of shift work systems in many countries [11]. Shift work is an organizational form of work devised to maintain production, transportation, health, or public order, requiring active workers around the clock [12]. These work schedules, including night shifts, have been associated with reduced sleep duration and quality, leading to other adverse health outcomes for workers [6].

Although night work has been associated with circadian misalignment, insufficient or poor-quality sleep, chronic fatigue, mental dysfunction, metabolic disturbances, and increased cardiovascular morbidity and mortality, there is a dearth of robust evidence in the literature on the association between night work exposure and arterial stiffness [12], one of the factors commonly involved in cardiovascular diseases.

There is a limited amount of research on arterial stiffness as a marker of vascular aging in shift workers, and no known studies have examined the association between arterial stiffness and social jet lag in this population. Additionally, the relationship between pulse wave velocity, an early indicator of arterial stiffness, and cardiovascular risk in shift workers has not been fully elucidated. Thus, the present study investigated the hypothesis that duration of exposure to night shifts is associated with arterial stiffness and explored the pathways between night work exposure and pulse wave velocity as a predictor of arterial stiffness in nursing professionals. To this end, the role of factors such as social jet lag, perceived stress, sleep duration and quality, and heart rate as mediators of this association were examined.

## 2. Results

The study participants were categorized based on their current exposure to night work (exposure/non-exposure). The mean age of participants was 44.38 ± 11.7 years. Most participants were female, had children, were married or in a stable relationship, and had completed graduate degree education. The total sample had a prevalence of obesity of 37.50%, and a mean body mass index (BMI) of 27.93 ± 6.2 Kg/m^2^. Regarding weight and BMI, there was no statistically significant difference between daytime and nighttime workers (Table 1).

The comparison of the groups revealed statistically significant differences for the variables having children (*p* = 0.05) and number of children (*p* = 0.01), as shift workers had a higher number of children than daytime workers. However, daytime workers had a higher level of education (*p* = 0.05) (Table 1).

Regarding health conditions, most study participants were non-diabetic (91.25%), non-dyslipidemic (70.0%), and non-hypertensive (81.25%). Concerning lifestyle habits, the majority of the assessed professionals were non-smokers (97.5%), physically inactive (63.75%), and alcohol consumers (51.25%) (These data are presented in Table S1 of the Supplementary Material).

**Figure 1:**
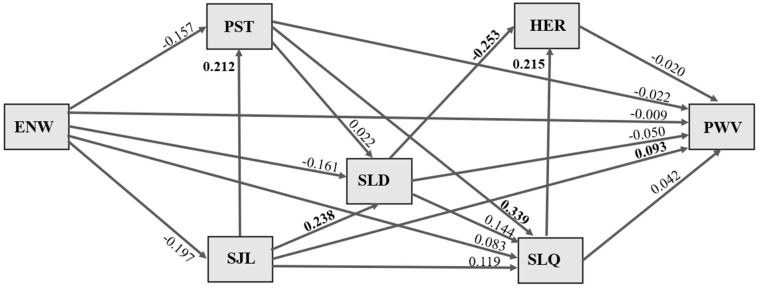
Path diagram for effects of length of exposure to night work (years) on pulse wave velocity. Values presented refer to standardized coefficients of associations tested, with items reaching statistical significance highlighted in bold. Analyses were adjusted for gender and age. Legend: ENW = exposure to night work, PST = perceived stress, SJL = social jet lag, SLD = sleep duration, HER = heart rate, SLQ = sleep quality, PWV = pulse wave velocity.

Most of the participants were nursing technicians (*p* < 0.001) (Table 2). Their mean duration of employment at the hospital was 9.20 ± 10.2 years, while their mean overall duration of exposure to night work was 7.79 ± 8.7 years. Their average working week was 51.05 ± 17.0 h and their mean time working the night shift was 18.86 ± 10.3 h per week. Most workers (58.75%, *n* = 47) held at least two jobs. Shift workers were predominantly nursing technicians/assistants, while day workers were mostly nurses (*p* < 0.001). It is worth noting that the data on average hours of exposure during the night shift are for the current exposure time, but in the path analysis (Figure 1), the inferential method was used, specifically lifetime exposure to night shift work, because changes related to vascular aging require exposure over time.

There were no statistically significant group differences in chronotype, social jet lag, number of awakenings, sleep efficiency, hemodynamic parameters, or arterial stiffness between night-shift and day workers (Table 3). The differences in social jet lag between the two groups observed in Table 3 refer to differences between means, while the path analysis is conducted in a way that shows the relationships between the variables.

The pathways for the effects of duration of exposure to night work on pulse wave velocity are illustrated in Figure 1. Values shown in bold refer to standardized association coefficients with statistical significance, and all associations were adjusted for sex and age. No significant indirect or direct effects of length of exposure to night work (years) on sleep duration, social jet lag, stress, sleep quality, heart rate, or pulse wave velocity were evident.

A direct effect of social jet lag on participants’ sleep duration was observed, whereby an increase of 1 standard deviation (SD) in social jet lag duration was significantly associated with a 0.238 SD increase in longer sleep duration (*p* = 0.030). An increase of 1 SD in social jet lag duration was significantly associated with a 0.212 SD increase in perceived stress (*p* = 0.047). An increase of 1 SD in social jet lag duration was significantly associated with a 0.093 SD increase in higher pulse wave velocity (*p* = 0.034).

A direct effect of stress on sleep quality was observed, whereby an increase of 1 SD in perceived stress scale score was significantly associated with a 0.399 increase (*p* = 0.000) in worse sleep quality. An increase of 1 SD in worse sleep quality was significantly associated with a 0.215 increase (*p* = 0.028) in higher heart rate. An increase of 1 SD in sleep duration was significantly associated with a 0.253 SD decrease in heart rate (*p* = 0.009).

## 3. Discussion

The results of this study showed no direct association between length of exposure to night work and arterial stiffness. However, path analysis revealed that greater social jet lag (SJL) was associated with higher pulse wave velocity (PWV), increased perceived stress, and longer sleep duration. Additionally, there was an association between higher perceived stress and worse sleep quality, nor between longer sleep duration and lower heart rate, among the healthcare workers studied.

In the study conducted by Haupt et al. [13], an association was observed between duration of exposure to night work and greater thickness of the carotid intima–media layer, also an indicator of arterial stiffness. However, this finding was not observed in the present study, as shift work was not associated with higher pulse wave velocity, a predictor of arterial stiffness. In another study, a significant association was found between shift work and increased carotid intima–media thickness in men, but not in women [14]. Given the majority of the present study population was female and no association was observed, this finding is consistent with previous reports.

Jankowiak et al. [15] reported that cumulative number of night shifts was associated with increased arteriosclerosis, evidenced by increased arterial stiffness measured by PWV and decreased vascular function estimated by the reactive hyperemia index. This finding differs from the results of the present study, identifying no positive association between duration of exposure to night work in years and higher PWV. In contrast, Silva-Costa et al. [16] showed an association between duration of exposure to night work and increased carotid intima–media thickness, an indicator of subclinical atherosclerosis, among men only. Thus, this article cited found no association in women, only in men, consistent with the results of the current study.

The difference in social jet lag between shift and day workers was small (0.41 vs. 0.48 h) and was not statistically significant. The path analysis, however, was conducted in a way that shows relationships between variables. Thus, we believe that, over the long term, with years of exposure to night work, social jet lag may indeed affect sleep timing and/or quality and, subsequently, PWV.

We understand that the numerical difference in PWV values between shift and day workers studied is not large, and that the way to interpret the path analysis is not straightforward. However, since this is an attempt to understand the higher prevalence of cardiovascular disease in workers exposed to night shifts, it is important to search mediators that can be involved in the process. It may not be of clinical relevance in this sample, but it is a strategy that can help to understand possible potentiators of the risk of premature vascular aging over the years of exposure to night work.

Although no direct association between duration of night work exposure and PWV was detected in the present investigation, exposure to night shifts has been recognized as a factor associated with increased SJL in workers [8]. Increased social jet lag was also associated with higher perceived stress which, in turn, was linked to worse sleep quality. Puttonen et al. [17] argued that psychosocial work stress and the imbalance between work and family life can be considered pathways underlying the association between shift work and subclinical atherosclerosis.

Studies have shown a significant association between short sleep during workdays and higher perceived stress in workers from South Korea [18] and Japan [19]. In contrast to the present study, Adachi et al. [20], who evaluated only day workers engaged in various occupations at a university, found no significant associations between social jet lag and stress. The biological mechanism of the relationship between social jet lag and stress is not fully elucidated, but it is known that circadian misalignment can negatively impact individuals’ psychological well-being and behavior [7]. However, while social jet lag was associated with stress in the current study, no association between stress and high PWV was evident. Therefore, it cannot be concluded that stress was a predictor of arterial stiffness in the individuals assessed.

In this study, increased social jet lag was also associated with longer sleep duration, which may seem paradoxical. A randomized, double-blind, crossover, controlled clinical trial found that nurses on a fixed night shift had shorter nighttime sleep duration and higher social jet lag [21], reinforcing that greater social jet lag is more commonly associated with shorter sleep duration.

A possible explanation for the association between increased social jet lag and longer sleep duration could be related to the rebound effect on off days. It is important to note that, in the present study, sleep duration included both working and non-working days, so this finding may reflect the increase in sleep duration on days off. From a cardiovascular risk perspective, although sleep duration did not directly mediate significant changes in PWV, there is evidence in the literature linking both short [22] and long [23] sleep duration to a higher risk of cardiovascular morbidity and mortality.

This analysis also showed that worse sleep quality was associated with increased heart rate. Similarly, reduced sleep duration was found to be associated with elevated heart rate. Sajjadieh et al. [24], in their study of the relationship of sleep duration and quality with blood pressure (BP) and heart rate variability (HRV), found that worse sleep quality was associated with higher heart rate and elevated systolic BP.

A study of nurses conducted by Hsu, Lee, and Lin [25] revealed that poor sleep quality was associated with alterations in HRV, indicating cardiovascular autonomic imbalance with increased sympathetic activation and higher heart rate. However, stress, including that caused by sleep restriction, can lead to autonomic dysfunction, with the reduced modulation of parasympathetic activity, potentially impacting HRV and increasing the heart rate [24]. Therefore, there is biological plausibility for the associations found in the current study, especially since heart rate tends to decrease during sleep, accompanying the rhythms of body temperature and metabolism.

Stress and anxiety at work have been linked to SJL, poor sleep duration, and cardiometabolic risk factors. A study conducted by Sládek et al. [26] on adults in the Czech population observed that social jetlag greater than or equal to 0.65 h was significantly associated with an increase in total cholesterol and low-density lipoprotein cholesterol, especially in those over 50 years of age. The findings of the present study corroborate the previous ones, as we observed in shift workers an association between JSL and PWV, an indicator of arterial stiffness and an independent predictor of cardiovascular risk. However, the jetlag of night workers may not have been sufficiently different to that of day workers to be directly associated with the night shift.

The cross-sectional design of this study precludes any inference on causal relationships. The number of participants included in the study is representative of the hospital’s nursing team and has a sampling power of 85%, which is sufficient to support the hypothesis. However, the sample was composed by convenience and almost exclusively of women, which does not allow for generalizations of the study findings. Another limitation was that some variables (alcohol consumption, physical activity, and being a current smoker) were presented in a dichotomized form, as they were obtained through closed questions. Since the study participants were members of the nursing team who answered the questionnaires during their work hours, the shorter and more objective the questions, the greater the likelihood of participation. The hemodynamic and arterial stiffness parameters were assessed only once a day, which can also be considered a limitation of the study. However, it is important to note that the outcome indicator investigated, pulse wave velocity, does not exhibit significant circadian variation. However, study strengths include the evaluation of central hemodynamic parameters and arterial stiffness (predictors of early cardiovascular risk), and use of actigraphy as a means of directly investigating the sleep onset time, sleep end time, number of awakenings, sleep duration, and sleep efficiency of the healthcare workers. Furthermore, detailed information on current work schedules, with clear descriptions of all employment relationships, working hours, and current night-work exposure, was collected. Data on past night-work exposure were also gathered.

It should be pointed out that, in the path model proposed, some potential mediation may not have been taken into account, possibly influencing the associations established. Additionally, the healthy worker effect (a special type of selection bias, as active workers, especially in night shifts, tend to be healthier than daytime workers or even the general population) may have weakened the power of the associations identified. Few studies on night shifts and arterial stiffness assessed using PWV were found in the literature, and no publications using path analysis were identified, hampering any meaningful comparison of results. It is important that more studies with a longitudinal design and with larger numbers of participants are conducted to investigate the association between exposure to night work, social jetlag, and arterial stiffness.

## 4. Materials and Methods

### 4.1. Design, Sampling, and Grouping

The cross-sectional observational study was conducted within a psychiatric hospital in the State of Alagoas, Brazil, and participants were recruited from all units of the institution that had nursing professionals on their staff.

Subjects that were nursing professionals, aged 18 years or older, currently working at the institution, and who agreed to participate, were included in the study. Professionals with any condition that could independently affect arterial stiffness parameters, such as pregnancy, lactation, chronic kidney disease etc., were not selected for the study.

The sample was non-probabilistic and convenience-based. Out of the total 121 nursing team professionals active at the hospital during the data collection period, 97 (80.17%) worked shifts and, of this group, 64 (65.98%) agreed to participate in the study. The sample size was calculated a priori, for a two-tailed proportion test to compare two independent groups (z-test for proportions). The meta-analysis conducted by Khoshdela et al. [27] was considered, which observed the prevalence of increased PWV ranging from 34.3% to 46.3% in individuals with moderate cardiovascular risk (i.e., those with one or more associated risk factors, but without the occurrence of an adverse cardiovascular event). Thus, the effect size was estimated at 40% (*p*_1_ = 0.40) for the night shift group and 10% for the day shift (control) group (*p*_2_ = 0.10), at 80% statistical power and the 5% significance level (α). The calculation indicated that 36 participants per group, totaling 72 participants, would be necessary to detect a reduction in prevalence with 80% power and 5% significance level. Furthermore, a possible attrition rate of 15% was considered, bringing the total to 83 participants.

However, significantly more night-shift workers agreed to participate in the study, while this was not the case for day-shift workers. Given that the groups ended up with different sample sizes and a different analysis was used, we calculated the power of the sample a posteriori. Considering 83 participants, six predictors, and the same parameters as before (*p*_1_ = 0.40; *p*_2_ = 0.10), the power of the sample for a linear multiple regression analysis was 94%. The sample calculation was performed using the statistical software G*Power 3.1.9.7. To achieve the objective of the study, only shift workers were included in the path analysis model. Of the 64, one participant was not included in the analyses because their actigraphy data were not recorded by the device. Thus, the final sample consisted of 63 shift-working professionals.

### 4.2. Data Collection

Data collection took place from September to November 2021. The data collection form included questions about sociodemographic profile, health status, lifestyle habits, work characteristics. The variable “exposure to night work” represented the total duration of exposure to night work throughout professional life, measured in years, and multiplied by the number of night hours worked per week throughout life.

Anthropometric nutritional status assessment was performed by measuring weight and height, as recommended in the literature. Based on these data, the Body Mass Index (BMI) was calculated and classified according to the cutoff points recommended by the World Health Organization [28].

Stress level was determined using the Perceived Stress Scale (PSS), a 14-item instrument, answered on a Likert-type frequency scale ranging from “never” (0) to “always” (4), with higher scores indicating higher perceived stress [29]. Sleep quality was assessed using the Pittsburgh Sleep Quality Index (PSQI), which contains 19 questions and consists of seven subscales: subjective sleep quality, sleep latency, sleep duration, sleep efficiency, sleep disturbances, use of sleep medication, and daytime dysfunction. Global PSQI score ranges from 0 to 21, with a score ≥ 5 indicating poor sleep quality [30].

In this study, participants’ chronotypes were assessed using the Munich ChronoType Questionnaire for shift-workers (MCTQShift), which collects sleep duration and timing data specific to different shifts. Unlike the standard MCTQ, which differentiates only between workdays and free days, the MCTQShift records mid-sleep times separately for each shift and its corresponding free days. Chronotype was determined based on mid-sleep between two free days after an evening shift block (MSFE). Additionally, shift-specific social jet lag was calculated as SJLx = MSWx − MSFx, following Juda et al. [31]. Sleep–wake patterns were assessed using actigraphy over a 10-day period using the ActTrust@ device (Condor Instruments, São Paulo, Brazil), where variables collected included sleep onset time, sleep end time, number of awakenings, sleep duration, and sleep efficiency.

Hemodynamic parameters of peripheral and central blood pressures and arterial stiffness (pulse wave velocity) were obtained using a non-invasive method. To ensure collection uniformity and maintain a standard regarding work exposure and biological rhythms, the tests were conducted on workdays in the afternoon, between 14:00 and 18:00 h. Prior to the hemodynamic assessment, participants were asked to empty their bladder, avoid physical activity in the preceding 60 min, and refrain from consuming food, coffee, alcoholic beverages, or smoking in the 30 min before testing. Measurements were taken with participants seated in a comfortable position within a quiet environment for 5 min [32]. The Arteris AOP^®^ oscillometric device (Cardios Sistemas Comercial e Industrial Ltd.a., São Paulo, Brazil) determines blood pressure based on the monitoring of brachial pulse waves (ARCsolver@ method, Austrian Institute of Technology, Giefinggasse, Vienna, Austria). To obtain central parameters, the device maintains inflation for 10 s at the level of diastolic brachial blood pressure, where a high-fidelity pressure sensor (MPX50550, Freescale Inc., Tempe, AZ, USA) captures the influence of arterial impedance using a transfer function, as well as aortic hemodynamics using a mathematical method [33].

The Arteris Automated Office Blood Pressure (AOP)^®^ employs the triple Pulse Wave Amplitude (PWA) technique, in which four automatic shots are taken with pre-established intervals of one minute between measurements, with the last three measurements considered valid for establishing hemodynamic parameters. This method allows the analysis of peripheral and central diastolic and systolic blood pressures, peripheral and central pulse pressures, heart rate, pulse wave velocity (PWV), and AIx@75, among other parameters. Participant data were classified according to normative reference values for the Brazilian population [32].

### 4.3. Data Analysis and Treatment

Data were tabulated in Excel 2013 (Microsoft Corporation, Redmond, WA, USA) independently by two researchers and, after checking for discrepancies, the tabulation was consolidated. For descriptive analyses, categorical variables were expressed as percentages, and quantitative variables as mean and standard deviations (SD). The adherence of continuous variables to a normal distribution was also assessed using the Shapiro–Wilk test. Path analysis was employed to test the relationship between length of exposure to night work (years) and pulse wave velocity (PWV). Unlike traditional regression analyses, path analysis is a method that uses multiple linear equations to include direct and indirect effects.

Based on the literature, models incorporating the following variables in the path analysis examining the association between length of exposure to night work (ENW) and pulse wave velocity (PWV) were tested: perceived stress (PST), social jet lag (SJL), sleep duration (SLD), heart rate (HER), and sleep quality (SLQ). The relationships between these variables were explored based on the fact that exposure to night work alters biological rhythms, which in turn can trigger social jet lag [7] and modify perceived stress [29]. Moreover, working at night is recognized as a factor influencing sleep duration and quality [34,35], both of which can impact the functions of the autonomic nervous system, modifying heart rate and altering pulse wave velocity [36]. Blood pressure, which affects vessel distensibility, was included as a confounding variable in the statistical model. However, the strength indices of the path analysis were not properly adjusted. Consequently, sex and age, which directly influence pulse wave velocity (PWV), were used as adjustment variables in the model [32].

**Figure 2:**
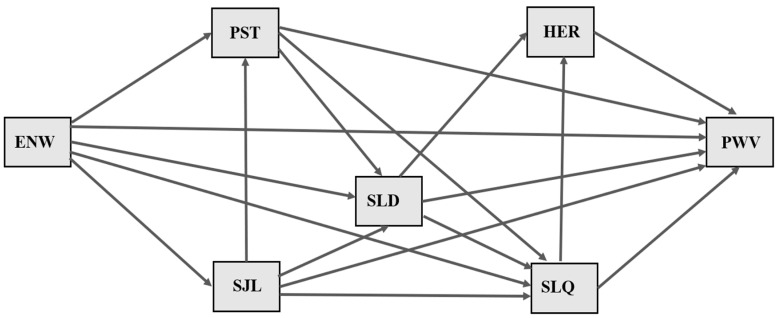
Estimated model for association between length of exposure to night work and pulse wave velocity using path analysis. Legend: ENW = exposure to night work, PST = perceived stress, SJL = social jet lag, SLD = sleep duration, HER = heart rate, SLQ = sleep quality, PWV = pulse wave velocity.

Some of the variables assessed were not included in the model for the following reasons: (1) having children, number of children, marital status, and education—lacking recognized biological plausibility in the genesis of arterial stiffness; (2) health status, lifestyle habits, and anthropometric nutritional status—having been associated with PWV in different studies and not constituting the central focus of this study. Additionally, most participants had a BMI ≥ 25 kg/m^2^, were not physically active, and not diabetic, hypertensive, or dyslipidemic, indicating the low discriminatory power of the variables; (3) work characteristics—used to elucidate participants’ job roles and total workload, but not directly related to the research question; (4) chronotype—used to calculate social jet lag; (5) number of awakenings and hemodynamic patterns—tested in the initial hypothetical model, but not retained in the final model due to poor statistical fit in the path analysis. The final estimated model is depicted in Figure 2.

The model estimated standardized coefficients, representing the relationship between explanatory and response variables, expressed as standard deviation (SD) units. The term “effect” refers to the association between the variables in the model, since the cross-sectional nature of this study precludes inferences on the direction of causality of the relationships.

The Maximum Likelihood (ML) estimation method was used to estimate the parameters. The model’s fit was determined using the Comparative Fit Index (CFI ≥ 0.90), Tucker–Lewis Index (TLI ≥ 0.90), Root Mean Square Error of Approximation (RMSEA ≤ 0.06), 90% Confidence Interval (90% CI), and Standardized Root Mean Square Residual (SRMR ≤ 0.08).

Standardized regression coefficients and their respective confidence intervals (95% CI), as well as model fit, were produced using Mplus software (version 7.4, Muthen & Muthen, Los Angeles, CA, USA). All descriptive analyses were performed using R Development Core Team software (version 4.1.2, R: A language and environment for statistical computing, 2021). Normality plots were generated using the ggplot2 and ggpubr R packages. The t-test, Wilcoxon’s test, and Pearson’s chi-squared test were used to compare parameters between shift and day workers, with a significance level set at 5%.

### 4.4. Ethical Considerations

The study was submitted to the Research Ethics Committee of the School of Public Health at the University of São Paulo (USP) and the State University of Health Sciences of Alagoas (UNCISAL). The study was approved by these committees in March 2021, under permit no. 4,607,182, and in May 2021, under permit no. 4,728,413, respectively. After approval, the researchers visited the work areas on various days and at different times, in accordance with the hospital’s work schedule, to individually invite professionals to participate in the study. Individuals that agreed to take part and gave consent by signing the Free and Informed Consent form were included in the study. The anonymity and confidentiality of the identity of the nursing team professionals were guaranteed.

## 5. Conclusions

This study found no direct association between the duration of night shift work exposure and arterial stiffness. Nonetheless, an association was identified between increased social jet lag and elevated PWV, an independent predictor of increased cardiovascular risk and target organ damage, in the workers studied.

## Figures and Tables

**Table 1:** Sociodemographic characteristics and anthropometric nutritional assessment of study participants.

Variables	Total	Night Work		
	(*n* = 80)	Yes (*n* = 63)	No (*n* = 17)	*p*-Value
Sociodemographic characteristics				
Age, years	44.38 ± 11.7	44.98 ± 10.5	42.12 ± 15.5	0.48
Sex, %				
Female	91.25	88.89	100.00	0.87
Male	8.75	11.11	0.00	
Have children, %	78.75	84.13	58.82	0.05 *
Number of children	1.62 ± 1.3	1.79 ± 1.3	1.00 ± 1.0	0.01 *
Marital status, %				
Single	30.00	25.40	47.06	0.40
Married/stable union	51.25	50.79	52.94	
Separated/divorced	13.75	17.46	0.00	
Widowed	5.00	6.35	0.00	
Education, %				
Complete high school	31.25	38.10	5.88	0.05 *
Complete undergraduate degree	16.25	14.29	23.53	
Incomplete graduate degree	5.00	4.76	5.88	
Complete graduate degree	36.25	30.16	58.82	
Anthropometric nutritional assessment				
BMI, Kg/m^2^	27.93 ± 6.2	28.04 ± 6.0	27.53 ± 6.9	0.62
Obesity, %				
Yes	37.50	36.51	41.18	0.94
No	62.50	63.49	58.82	

Legend: BMI = body mass index. Note: the tests used to compare groups were the *t*-test, Wilcoxon’s test, and Pearson’s chi-squared test. * denotes values considered statistically significant (*p* ≤ 0.05).

**Table 2:** Work-related characteristics and the perceived stress of the participants.

Variables	Total	Night Work		
	(*n* = 80)	Yes (*n* = 63)	No (*n* = 17)	*p*-Value
Job position, %				
Nurse	32.50	20.63	76.47	*p* < 0.001 *
Nursing Technician/Assistant	67.5	79.36	23.53	
Time at institution, years	9.20 ± 10.2	9.43 ± 9.6	8.36 ± 12.4	0.21
Working week, hours	49.10 ± 16.7	51.05 ± 17.0	41.88 ± 13.3	0.07
Night work, h/week	-	18.86 ± 10.3	0.00 ± 0.0	-
Exposure to night work, years	-	7.79 ± 8.7	0.41 ± 1.7 ^1^	-
Perceived Stress Scale				
Mean score	24.27 ± 9.0	23.73 ± 9.2	26.29 ± 8.4	0.28
Sleep quality—poor, %				
Yes	83.75	80.95	94.12	0.34
No	16.25	19.05	5.88	

Note: the tests used to compare the groups were the *t*-test, Wilcoxon’s test, and Pearson’s chi-squared test. ^1^ Some participants had previously worked nights for short stints during their professional life but had stopped at least 2 years before their interview. * denotes values considered statistically significant (*p* ≤ 0.05).

**Table 3:** Characteristics regarding temporal preference, sleep–wake patterns, hemodynamic parameters, and arterial stiffness of participants.

Variables	Total	Night Work		
	(*n* = 80)	Yes (*n* = 63)	No (*n* = 17)	*p*-Value
Temporal preference				
Chronotype, h	27.32 ± 1.5	27.29 ± 1.5	27.46 ± 1.6	0.68
Social jet lag, h	0.43 ± 1.0	0.41 ± 1.0	0.48 ± 0.7	0.57
Sleep–wake patterns				
Number of awakenings, n.	9.07 ± 3.7	8.75 ± 3.6	10.29 ± 4.2	0.17
Sleep duration, h	5.54 ± 0.8	5.48 ± 0.9	5.78 ± 0.5	0.07
Sleep efficiency, %	83.72 ± 5.8	83.73 ± 6.0	83.68 ± 5.0	0.97
Hemodynamic parameters and stiffness				
Peripheral systolic pressure, mmHg	111.71 ± 12.2	112.21 ± 12.2	109.0 ± 12.3	0.49
Peripheral diastolic pressure, mmHg	79.85 ± 9.9	80.62 ± 9.0	77.00 ± 12.7	0.73
Central systolic pressure, mmHg	104.97 ± 11.8	105.67 ± 12.0	102.41 ± 11.0	0.29
Central diastolic pressure, mmHg	80.86 ± 9.9	81.59 ± 9.1	78.18 ± 12.6	0.78
Heart rate, bpm	75.28 ± 13.2	74.11 ± 13.6	79.59 ± 10.8	0.20
Pulse wave velocity, m/s	6.32 ± 1.4	6.35 ± 1.2	6.22 ± 1.8	0.22
Augmentation index (AIx@75), %	19.56 ± 8.7	19.73 ± 9.1	18.94 ± 7.3	0.71

Legend: AIx@75 = Augmentation index corrected for a heart rate of 75 bpm. Note: the tests used to compare groups were the *t*-test, Wilcoxon’s test, and Pearson’s Chi-squared test. The path analysis model presented in this article pertains to the relationships between exposure to night work, social jet lag, perceived stress, sleep duration, heart rate, sleep quality, and pulse wave velocity. Based on the reference criteria outlined in the Methods section, this model demonstrated a high quality of fit (Root Mean Square Error of Approximation—RMSEA = 0.035, Comparative Fit Index—CFI = 0.997, Tucker–Lewis Index—TLI = 0.983, and Standardized Root Mean Square Residual—SRMR = 0.045).

## Data Availability

The data presented in this study are available on request from the corresponding author due to restrictions of privacy and ethical reasons.

## Supplementary Materials

The following supporting information can be downloaded at: https://www.mdpi.com/article/10.3390/clockssleep7010010/s1. Table S1: Health status and lifestyle habits of study participants.
