# Supplementary material for: Night Work and Social Jet Lag: Pathways to Arterial Stiffness?
Source: Clocks Sleep. 2025 Mar 3;7(1):10. doi: 10.3390/clockssleep7010010 (PMC11941686; doi:10.3390/clockssleep7010010)
Supplement: Supplementary file 1 [file clockssleep-07-00010-s001.zip › clockssleep-3426956-supplementary.pdf]

Supplementary material. Health status and lifestyle habits of study participants.

Variables	Total	Night Work		
	(n=80)	Yes (n=63)	No (n=17)	p-value
Diabetes mellitus, %				
Yes	8.75	9.52	5.88	
No	91.25	90.48	94.12	1.00
Dyslipidemia, %				
Yes	30.00	28.57	35.29	
No	70.00	71.43	64.71	0.81
Use of antihypertensive medication, %				
Yes	18.75	19.05	17.65	
No	81.25	80.95	82.35	1.00
Alcohol consumption, %				
Yes	51.25	52.38	47.06	
No	48.75	47.62	52.94	0.90
Current smoker, %				
Yes	2.50	3.17	0.00	
No	97.5	96.83	100.0	1.00
Physically active, %				
Yes	36.25	36.51	35.29	
No	63.75	63.49	64.71	1.00

Note: Tests used to compare groups were the t-test, Wilcoxon's test, and Pearson's chi-squared test.

* Values considered statistically significant (p≤0.05).
